# Supplementary material for: Receiving quality antenatal care service increases the chance of maternal use of skilled birth attendants in Ethiopia: Using a longitudinal panel survey
Source: PLoS One. 2022 Dec 22;17(12):e0279495. doi: 10.1371/journal.pone.0279495 (PMC9778501; doi:10.1371/journal.pone.0279495)
Supplement: Attachment — Submitted filename: comment to authors.docx [file pone.0279495.s001.docx]

Title: Receiving quality antenatal care service increases the chance of maternal use of skilled birth attendants in Ethiopia: Using a longitudinal panel survey

Dear authors

This is interesting research area. I found the question authors raised very important. Coverage of antenatal care alone couldn’t bring required skilled birth attendance. The coexistence of low quality and coverage ANC in Ethiopia and LMICs, has been obstacle to improve maternal and child health for decades. However, I have also issues to be addressed in the new version of the manuscript. Please, find the comments below:

The authors need be consistent while using terms throughout the manuscript. For instance, postnatal vs postpartum, mothers’ vs women, health care providers vs health professional, skilled birth attendant vs skilled workers, antenatal care vs prenatal care, ANC component quality vs ANC quality etc.

Background

Is quality ANC guarantee to use skilled birth attendant? What is an evidence in high-income countries?

What really contributed to low skilled birth attendant utilization for a women who had at least one ANC visit? Do you think your design is appropriate to explore it?

Method

Study design

“Then, women expected date of delivery was estimated…” how do you overcome recall bias here?

Study population

Who are your source and study population? “All postnatal mothers who had any ANC visit during pregnancy,….” Do you mean all women who received ANC from skilled attendants? What about women who received ANC from other professionals?

Sampling technique

Check how sampling were conducted for panel study? How stratification is performed? “At the second stage, 35 households per cluster were selected and all pregnant women aged 15-49 in the selected households were enrolled and interviewed.”?

Data collection method

“In PMA-Ethiopia panel survey,…” what makes it panel? How many times did they collect the data? This part need detail explanation of tool, data collectors and techniques unless it has been explained elsewhere.

How did you make sure women delivered by skilled attendant (health professional with midwifery skills)? Do you think rural women can differentiate these health professionals? “did you deliver by a skilled attendant?” “type of professional skilled birth attendant”

People rarely measure quality due to its measurement issues; how did you measure quality of ANC? Can we say “quality ANC” by measuring the ANC components only? Have you tried to use model to measure quality? How do you see demand and supply side issues?

Have you considered weighting while calculating ANC quality score from these 13 variables? What will be your answer if reader ask you is it fair to give one for HIV test, taking stool sample, birth preparedness and complication readiness etc.?

Data management and analysis

What were the proportion of missing? How missing data were managed in this analysis?

Why weighting was conducted? How weighting was conducted?

Have checked multi-collinearity? For instance, ANC quality vs ANC4+

“….generalized estimating equation (GEE) Poisson regression model was fitted…” was there significant correlation? Do GEE account for significant correlation within clusters?

Result

Table 1- some category need categorizing, since the cell percentage is very small.

“Number of months pregnant women first talked to health care provider (<=4 months vs >4 months)” what do you mean? Do you mean time of first ANC initiation?

Please take the following to method section; “In this study, 13 major prenatal care services received by mothers were used to create antenatal care quality index from women’s perspective. Women were classified as received ‘better ANC quality’ if they received more than the 75th percentiles of ANC service.”

Type of service received: Have you tried principal component analysis?

No need of repeating the model in the result; “Generalized estimating equation Poisson regression model was used to assess the effect of ANC quality on maternal use of skilled delivery.”

What community level factors or individual level factors did you identify in your case?

Discussion

“Improving the ANC service delivery rather than focusing primarily on facility readiness (inputs) may increase women and partner perceived quality of care because use of services and outcomes are the result not only of the provision of care but also of women’s experience of that care.” what if the low quality ANC was due to inputs/facility readiness? Or what if skilled attendants failed to perform the ANC components due to supply side problems, knowledge, skills, attitude etc.?

What do ANC quality (54%) tell us? Nearly half of the participants received quality ANC….

What is the implication of your study?
